# Correction: Iron Supplementation Decreases Severity of Allergic Inflammation in Murine Lung

**DOI:** 10.1371/annotation/5e0195b6-60b9-4c03-84ae-c6c31e625be1

**Published:** 2014-01-30

**Authors:** Laura P. Hale, Erin Potts Kant, Paula K. Greer, W. Michael Foster

Panel A in Figure 1 is incorrect. Please see the corrected figure here: 

**Figure:**
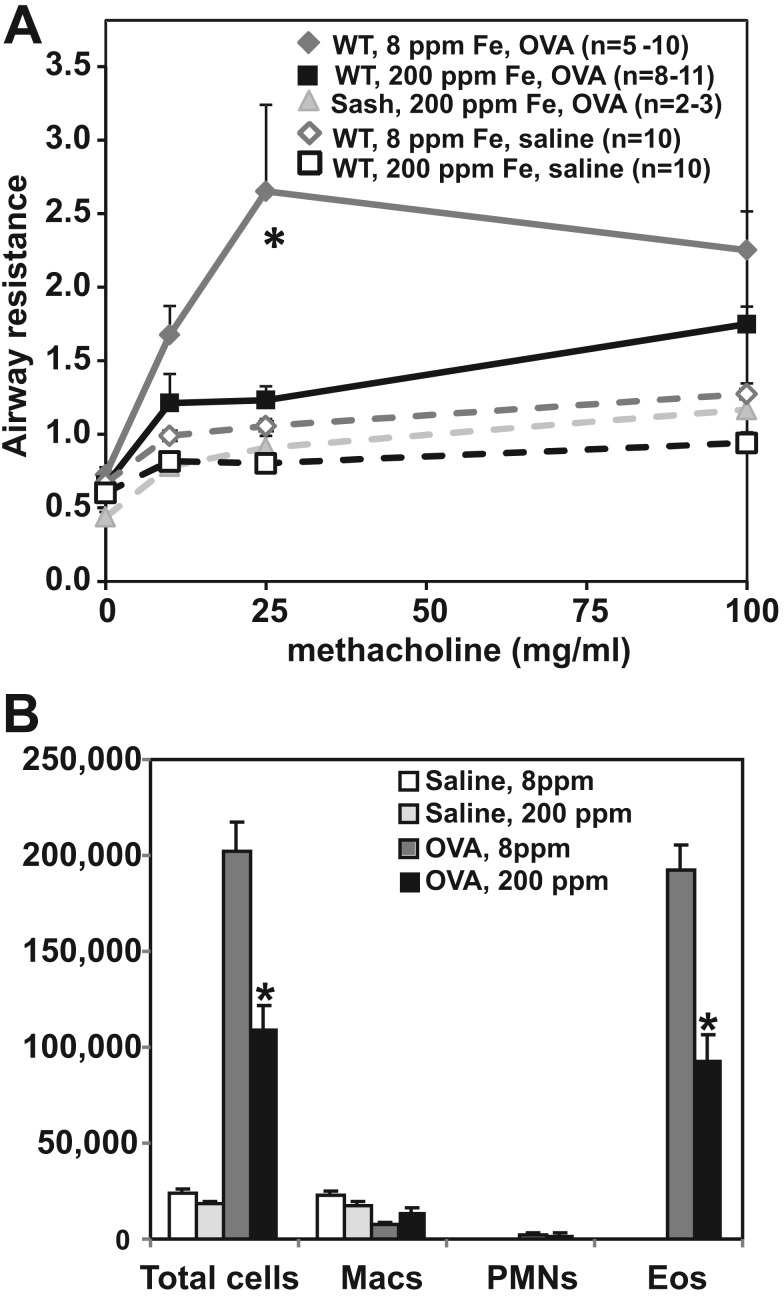


The incorrect p value is listed in the legend for Figure 1 and in the Results. The corrected p value should read "p<0.05" instead of "p<0.0005". The fifth sentence of the Results section should then read "These changes in airway resistance were markedly attenuated (38% reduction) in OVA-challenged mice on the iron supplemented (200 ppm) diet (Figure 1A; p< 0.05)".

